# Acute Recurrent Pancreatitis With Exclusion of Biliary Causes in a Young Female Patient

**DOI:** 10.7759/cureus.46246

**Published:** 2023-09-30

**Authors:** Jiayi Li, Thomas J Painter

**Affiliations:** 1 Molecular and Cellular Sciences, Liberty University College of Osteopathic Medicine, Lynchburg, USA; 2 Acute Care Surgery, Cheasapeake Regional Medical Center, Cheasapeake, USA

**Keywords:** mrcp, gallstone pancreatitis, sphincter of oddi dysfunction, cholesterolosis, idiopathic pancreatitis

## Abstract

Biliary pathologies are common causes of acute pancreatitis, including gallbladder cholesterolosis and gallstone pancreatitis. Nevertheless, after these two pathologies have been excluded, a broader differential and less common etiologies must be considered. Here we report an 18-year-old female with preliminary diagnosis of gallstone pancreatitis who underwent cholecystectomy and intraoperative cholangiogram resulting in uneventful recovery and resolution of symptoms. She returned three times within the month with symptoms of acute pancreatitis. We discussed alternative etiologies of her pancreatitis after exclusion of other causes with extensive imaging and laboratory evaluation.

## Introduction

The annual incidence of acute pancreatitis worldwide is increasing due to increased prevalence of gallstones and obesity. Gallstones account for 40-70% of all cases, especially in female patients as they are at higher risk for cholelithiasis [1]. Another benign condition that has been reported to cause acute pancreatitis is cholesterolosis of the gallbladder mucosa, which is the formation of cholesterol polyps that can break off and cause mechanical obstruction of the sphincter of Oddi (SO) [2]. Clinically, these two conditions can have similar presentations such as cholecystitis, pancreatitis, and biliary colic [2,3]. This is due to the shared pathophysiology of obstruction-type inflammation of the biliary-cystic-pancreatic duct system. The symptoms should completely resolve after cholecystectomy, removal of the obstructive source, and successful intra-operative cholangiogram, with clearance of any residual obstruction ensuring patent ductal anatomy. However, if the symptoms recur, it is critical to consider other etiologies.

## Case presentation

**Figure 1:**
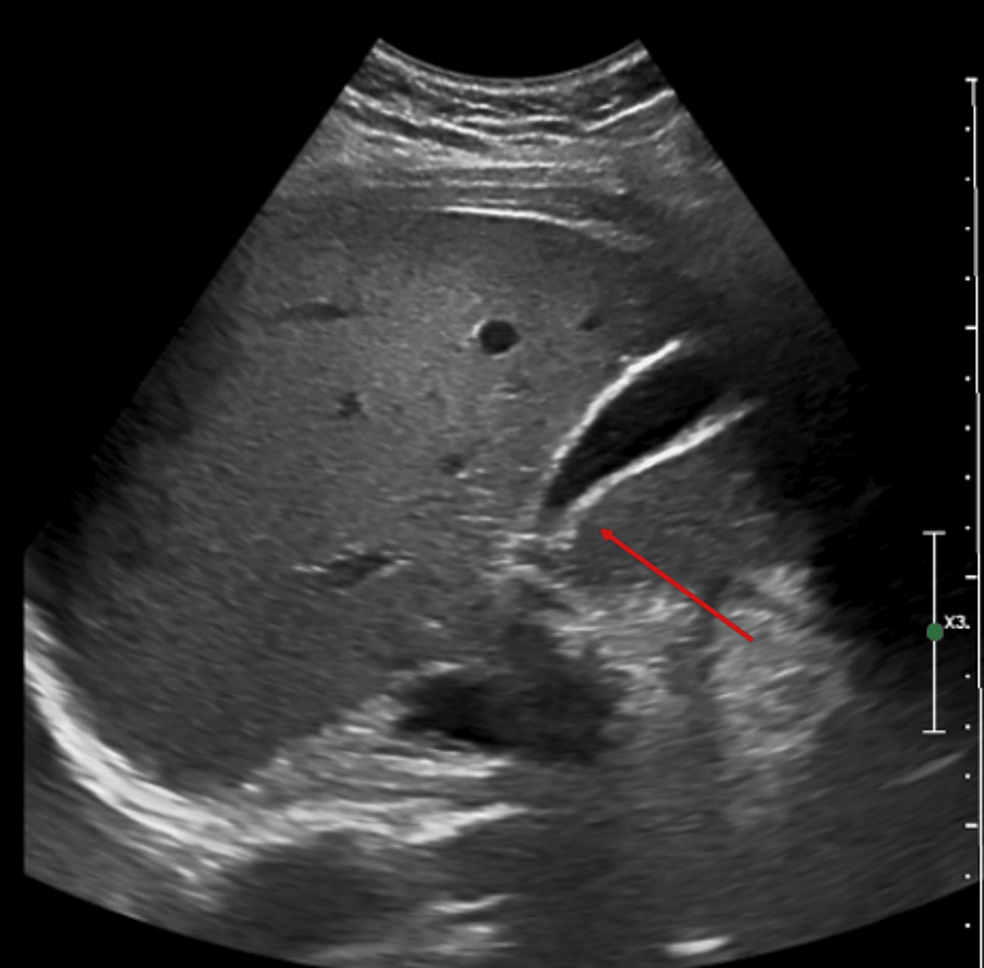
Ultrasonography shows normal gallbladder with no CBD dilation. CBD: common bile duct

**Figure 2:**
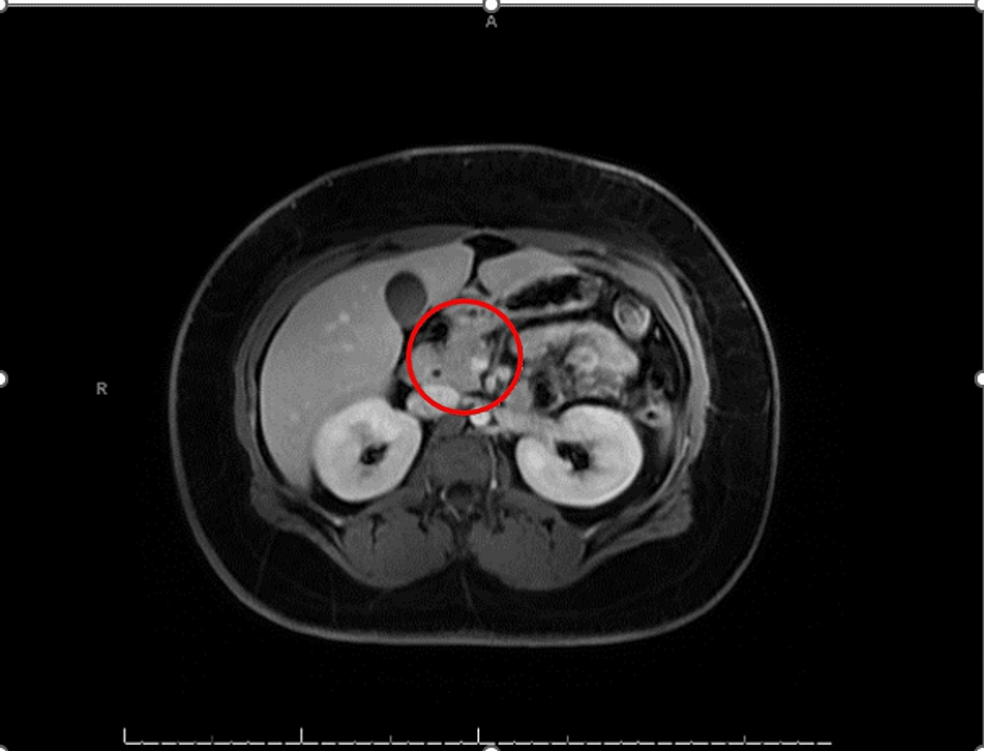
MRCP shows edematous changes and fat stranding around the head of the pancreas. MRCP: magnetic resonance cholangiopancreatography

**Figure 3:**
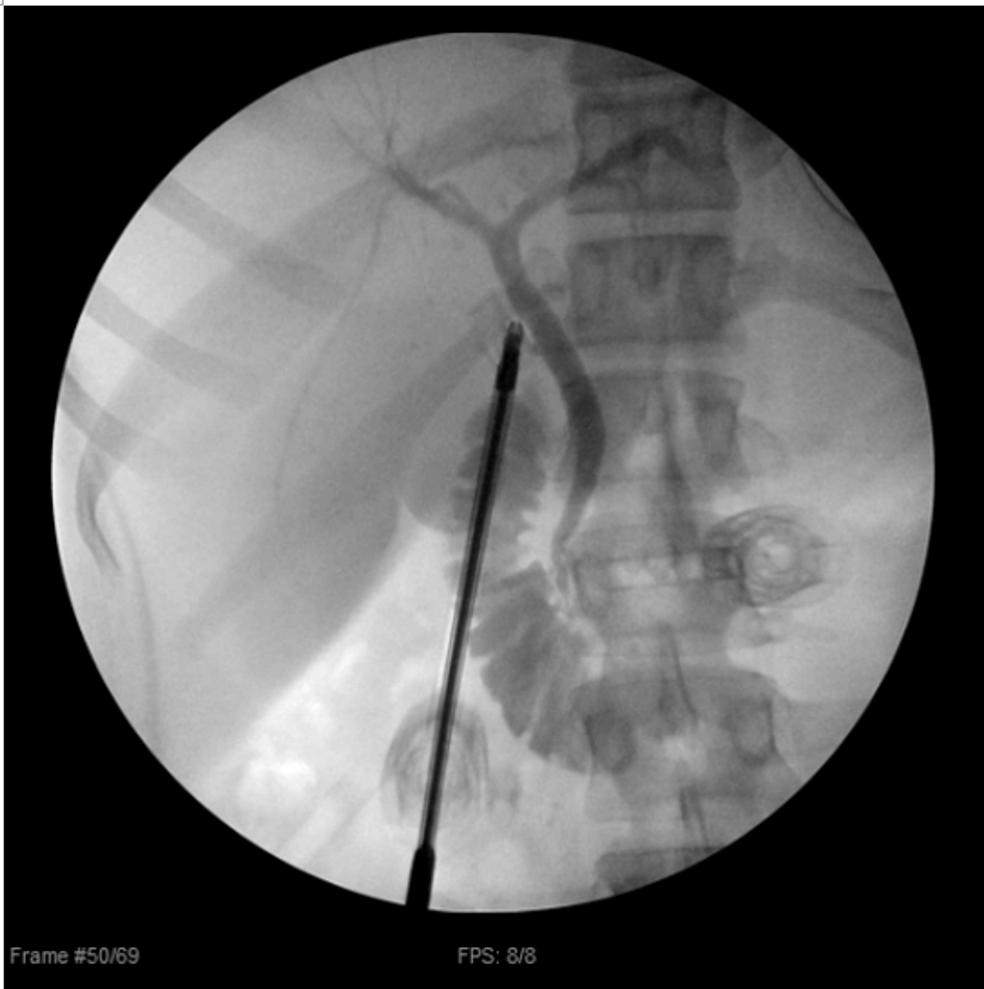
IOC demonstrating filling from the cystic duct to the biliary tree and duodenum without obvious obstruction. IOC: intraoperative cholangiogram

On 08/03/23, an 18-year-old female presented to the emergency department (ED) with moderate to severe pain in the epigastric and right upper quadrant for the past week. Her last meal before the pain consisted of yogurt and chicken salad. She had no significant past medical history and did not drink alcohol or use tobacco products. Her body mass index was 33.3. The only medication was a birth control patch. At ED admission, the patient had stable vital signs as follows: blood pressure 131/81, pulse rate 71 per minute, respiratory rate of 15, temperature of 97.5 °F (36.4 °C), oxygen saturation of 96%. Laboratory results were significant for elevated lipase and leukocytosis but the remainder, including hepatic panel, were normal (Table 1). Right upper quadrant ultrasound showed findings suggesting normal gallbladder without stones or sludge (Figure 1). A magnetic resonance cholangiopancreatography (MRCP) was obtained, with findings suggestive of acute pancreatitis with few mildly enlarged peripancreatic lymph nodes, possible biliary sludge, and no evidence of biliary duct dilatation or choledocholithiasis (Figure 2). On physical exam, her abdomen was non-distended, soft, mildly tender in epigastrium and right upper quadrant. A primary diagnosis of gallstone pancreatitis was made based on clinical presentation and imaging results. The patient was offered and consented for laparoscopic cholecystectomy with intraoperative cholangiogram (IOC) to rule out any potential obstructions not visualized on imaging. The surgery was uneventful with findings of chronic cholecystitis and steatosis in the liver. The cholangiogram demonstrated filling of the common bile duct (CBD) and bilateral hepatic ducts with flow into the duodenum without evidence of filling defects (Figure 3). Post-operatively, the patient reported improvement in abdominal pain with decreased lipase level, and the patient was discharged home on post-op day one.

**Table 1:** Hepatic profile and lipase levels throughout the initial two encounters, showing significantly decreased lipase level after the surgery then became elevated three days later. (Reference normal lipase level 10-140 U/L) Alk Phos: alkaline phosphatase, ALT: alanine aminotransferase, AST: aspartate aminotransferase

	8/4/2023	8/5/2023	8/8/2023	8/9/2023	8/10/2023	8/13/2023
Albumin	3.3	3.3	3.9	3.6	3.5	3.8
Alk Phos	77	85	100	95	95	99
ALT	7	40	38	30	27	24
AST	20	32	8	19	20	8
Bilirubin total	0.4	0.3	0.3	0.4	0.4	0.3
Lipase	518	72	576	432	336	280

**Figure 4:**
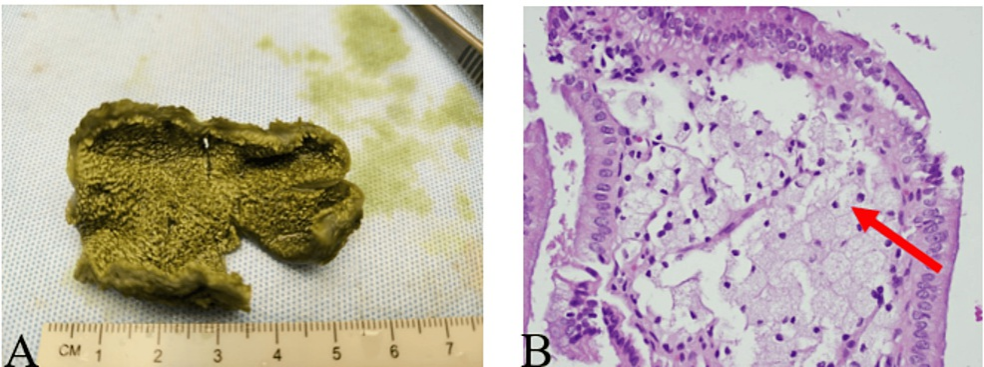
A. Gross appearance of the gallbladder mucosa showing diffuse yellow dots on the lining of the gallbladder, B. Photomicrograph at 40x magnification of gallbladder specimen demonstrating cholesterolosis with characteristic foamy macrophage (arrow).

The gallbladder measured 5.8x2.3x1.4 cm. The external surface was green tan and smooth. The content was dark green viscous bile. No calculi were identified in the lumen or specimen container. The gallbladder wall averaged 0.2 cm in thickness, and the mucosa was green tan and velvety. Histological examination with the pathologist revealed mild chronic cholecystitis with cholesterolosis (Figure 4).

**Figure 5:**
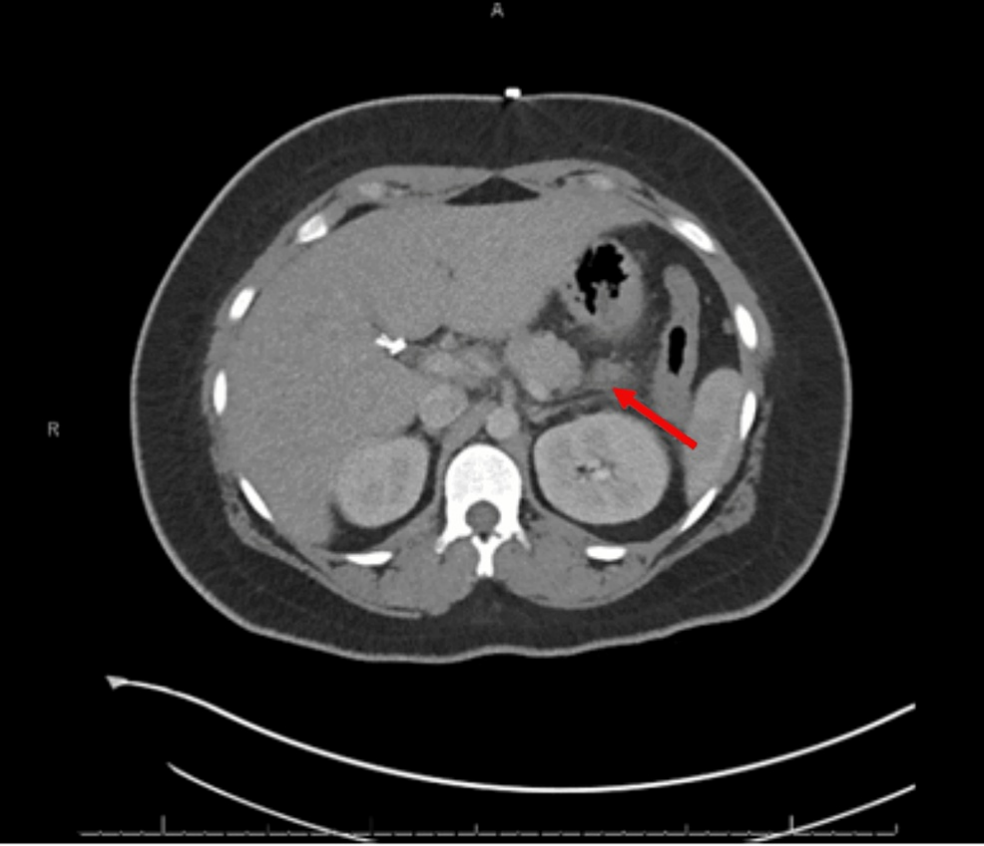
CT scan showing mild edematous changes in the tail of the pancreas. CT: computed tomography

**Figure 6:**
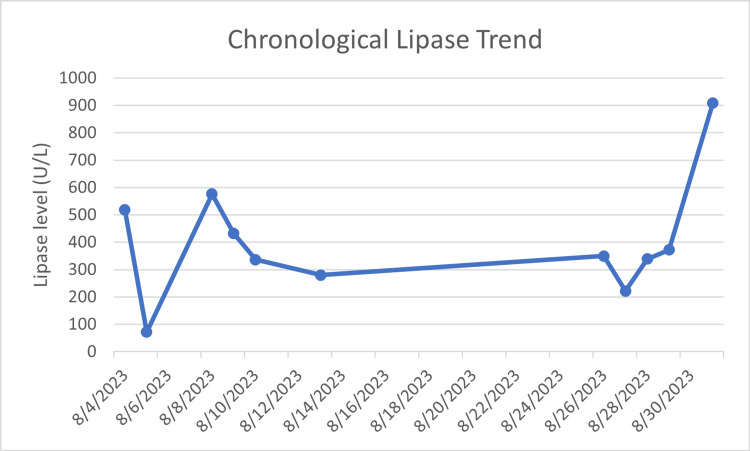
Chronological changes in the patient’s lipase level since initial admission followed by three subsequent admissions Reference range: 10-140 U/L

Three days after the patient was discharged home, she returned to the ED complaining of recurrent severe pain that was identical to her initial admission. She reported eating scrambled eggs a few hours before the onset of the pain. Upon presentation, her vitals were: temperature 98.1, blood pressure 161/88, and pulse rate of 83. On examination she had epigastric tenderness that radiated to her right flank. Computed Tomography (CT) of abdomen and pelvis demonstrated mild edematous changes in the tail of the pancreas, suggesting pancreatitis (Figure 5). Her lipase level was again elevated to 576, but hepatic panel remained normal. The patient was kept nothing by mouth (NPO), given intravenous (IV) fluid and analgesics as needed. Meanwhile, Gastroenterology ordered autoimmune pancreatitis workup, which came back with antinuclear antibody (ANA), anti-smooth muscle antibody (ASMA), and IgG4 level within normal range. The patient was slowly advanced to a low-fat diet and discharged home as her pain subsided. However, on 08/26, the patient returned to the ED with epigastric pain not relieved by non-steroid anti-inflammatory drugs (NSAIDs). She was given IV morphine as her symptoms improved and was discharged after three days of stay with ketorolac for pain control. On the morning of 08/31, she returned to the ED with severe pain that woke her up at night, and ketorolac provided no relief. Her lipase level was 909 U/L at this admission (Figure 6). At this point, a referral was made to a specialty pancreatitis center at an academic hospital per patient’s wish.

## Discussion

This patient’s recurrent pancreatitis post-op challenges the primary diagnosis of cholecystic origin. In many patients with acute idiopathic pancreatitis, cholecystectomy usually results in the resolution of symptoms. According to a recent systematic review and meta-analysis, the recurrence rate of pancreatitis is lower after cholecystectomy in patients with idiopathic acute pancreatitis [4]. However, in this case neither gallstone nor cholesterol polyp can cause irritation or obstruction of the pancreatic duct after the removal of the gallbladder with successful normal cholangiogram which was prompted by the possible sludge in MRCP. Cholesterolosis is typically a benign condition that has however been connected to acute pancreatitis [5]. In our patient, despite the diagnosis confirmed by microscopic findings, there were no appreciable polyps on imaging studies or gross examination of the specimen. This makes cholesterolosis as the etiology of her pancreatitis less likely. Obstruction of the common bile duct will likely also cause elevated hepatic panel results, which were never observed in this patient since the initial encounter. Retained gallstone after cholecystectomy can also result in pancreatitis, which is usually resolved with endoscopic retrograde cholangiopancreatography (ERCP), however the normal IOC would have ruled out any potential stone or sludge remaining in the biliary duct [6]. At this point, other less common etiologies should be considered. One of them is pancreatic type II sphincter of Oddi dysfunction (SOD). SOD is a condition characterized by motility dysfunction of the muscle that controls the flow of bile and pancreatic secretions into the small bowel. This condition falls under one of the early post-cholecystectomy syndromes, which is characterized by persistent biliary colic, right upper quadrant pain, or recurrent pancreatitis after the procedure [7]. The pain is presumed to be due to obstructed biliary or pancreatic flow leading to increased upstream pressure when there is SO smooth muscle dysmotility under the stimulation of cholecystokinin or secretin [8]. This is especially evident in the post-cholecystectomy population where removal of the gallbladder may eliminate a reservoir for backflow of bile resulting in more irritation of the pancreatic duct.

The Milwaukee Classification describes biliary and pancreatic as two main categories of SOD with three subtypes within each category based on clinical presentation, laboratory findings, and imaging [9]. Biliary SOD type I is characterized by biliary-type pain, elevated liver enzymes, and dilated CBD. Pancreatic SOD type I is diagnosed with pancreatic-type pain, amylase or lipase elevation, and dilated pancreatic duct. For both biliary and pancreatic SOD, type II requires pain symptoms with one or two of the objective findings, while type III has pain symptoms only. The gold standard for the diagnosis of SOD is sphincter of Oddi manometry (SOM) which shows elevated basal pressure (greater than 40 mmHg) of the biliary or pancreatic sphincter. A high incidence of abnormal SOM has been reported in idiopathic acute recurrent pancreatitis implying that a significant portion of patients likely have SOD and would benefit from treatment [10]. In patients with SO stenosis, the pressure measurement does not change with administration of muscle relaxants. In SO dyskinesia, there is decrease in pressure in response to muscle relaxant, excess retrograde contraction and paradoxical response (increase in pressure) to administration of cholecystokinin (CCK) [11]. However, the technical challenge, invasiveness and clinical complications encouraged development of other tools to evaluate. Currently, the least invasive way to diagnose a pancreatic SOD is through secretin-enhanced MRCP (S-MRCP), where secretin is given to stimulate dilation of pancreatic duct and SO to fill the duodenum with pancreatic secretions [12]. If the contrast fails to enter the duodenum or is severely delayed, the diagnosis is made. The treatment for this condition is biliary sphincterotomy alone or dual biliary and pancreatic sphincterotomy, with dual sphincterotomy resulting in a significantly better symptomatic improvement [13].

## Conclusions

Post-cholecystectomy idiopathic pancreatitis is a rare presentation that should raise red flags in clinical settings. It should challenge clinicians to reconsider the primary clinical diagnosis prior to the surgery, and prompt further workup for less common etiologies. Both the negative results for autoimmune pancreatitis and the minimum lipase value recorded after IOC peripherally suggest a sphincter pathology. Furthermore, her refractory pain regardless of dietary changes and NSAID use also corroborates with the spasmic pressure pain due to SOD. The normal hepatic panel throughout her stay makes the more common biliary SOD less likely. Overall, the clinical course, laboratory results, and imaging results for this patient with acute recurrent pancreatitis suggest pancreatic type II SOD as the probable etiology.

## References

[1] Yadav D, Timmons L, Benson JT, Dierkhising RA, Chari ST (2011). Incidence, prevalence, and survival of chronic pancreatitis: a population-based study. Am J Gastroenterol.

[2] Srivastava P, Srivastava S (2023). Strawberry gallbladder: a velvety carpet of surgical anguish. Gastroenterol Hepatol Open Access.

[3] Hines JH, Pillai S (2023). Gallstone pancreatitis post laparoscopic cholecystectomy: a case report. Cureus.

[4] Umans DS, Hallensleben ND, Verdonk RC (2020). Recurrence of idiopathic acute pancreatitis after cholecystectomy: systematic review and meta-analysis. Br J Surg.

[5] De Armas RE, Rosenberg JM, Fenves AZ (2018). Cholesterolosis as a cause of acute pancreatitis. Proc (Bayl Univ Med Cent).

[6] Anwar S, Rahim R, Agwunobi A, Bancewicz J (2004). The role of ERCP in management of retained bile duct stones after laparoscopic cholecystectomy. N Z Med J.

[7] Bistritz L, Bain VG (2006). Sphincter of Oddi dysfunction: managing the patient with chronic biliary pain. World J Gastroenterol.

[8] Villavicencio Kim J, Wu GY (2022). Update on sphincter of Oddi dysfunction: a review. J Clin Transl Hepatol.

[9] Geenen JE, Hogan WJ, Dodds WJ, Toouli J, Venu RP (1989). The efficacy of endoscopic sphincterotomy after cholecystectomy in patients with sphincter-of-Oddi dysfunction. N Engl J Med.

[10] Kaw M, Brodmerkel GJ Jr (2002). ERCP, biliary crystal analysis, and sphincter of Oddi manometry in idiopathic recurrent pancreatitis. Gastrointest Endosc.

[11] Toouli J, di Francesco V, Saccone G, Kollias J, Schloithe A, Shanks N (1996). Division of the sphincter of Oddi for treatment of dysfunction associated with recurrent pancreatitis. Br J Surg.

[12] Swensson J, Zaheer A, Conwell D, Sandrasegaran K, Manfredi R, Tirkes T (2021). Secretin-enhanced MRCP: how and why-AJR Expert Panel Narrative Review. AJR Am J Roentgenol.

[13] McLoughlin MT, Mitchell RM (2007). Sphincter of Oddi dysfunction and pancreatitis. World J Gastroenterol.

